# Porous Architecture–Driven Electroless Nickel Plating in Conventional and Pickering PolyHIPEs

**DOI:** 10.1021/acsomega.6c00968

**Published:** 2026-07-22

**Authors:** Nihan Sengokmen-Ozsoz, Enes Durgut, Frederik Claeyssens

**Affiliations:** † Department of Materials Science and Engineering, 52962 Gebze Technical University, Gebze, Kocaeli 41400, Turkey; ‡ Department of Genetics and Bioengineering, 450199 Alanya Alaaddin Keykubat University, Antalya 07425, Turkey; § Kroto Research Institute, School of Chemical, Materials and Biological Engineering, 7315 University of Sheffield, Sheffield S3 7HQ, U.K.

## Abstract

Polymerized high internal phase emulsions (polyHIPEs) offer highly porous and tunable architectures that are promising as templates for functional coatings. In this study, polyHIPE thin films were fabricated using either conventional surfactant-stabilized emulsions (Hypermer B246) or Pickering emulsions stabilized by isobornyl acrylate (IBOA) microparticles, and subsequently subjected to electroless nickel plating to evaluate the effect of emulsion stabilization on metal infiltration. SEM, EDX, and XRD analyses revealed that conventional polyHIPEs featured smaller, more uniform pores, resulting in surface-localized nickel deposition due to rapid pore closure. In contrast, Pickering polyHIPEs displayed larger, irregular pore structures. Although these structures exhibited restricted interconnectivity compared to conventional foams, their macroscopic pore dimensions facilitated deep mass transport, enabling uniform internal metallization. These results highlight that pore size magnitude can override interconnectivity limitations in diffusion-controlled plating processes, offering critical design insights for developing metal–polymer hybrids for applications such as catalysis and Electromagnetic Interference (EMI) shielding.

## Graphical abstract

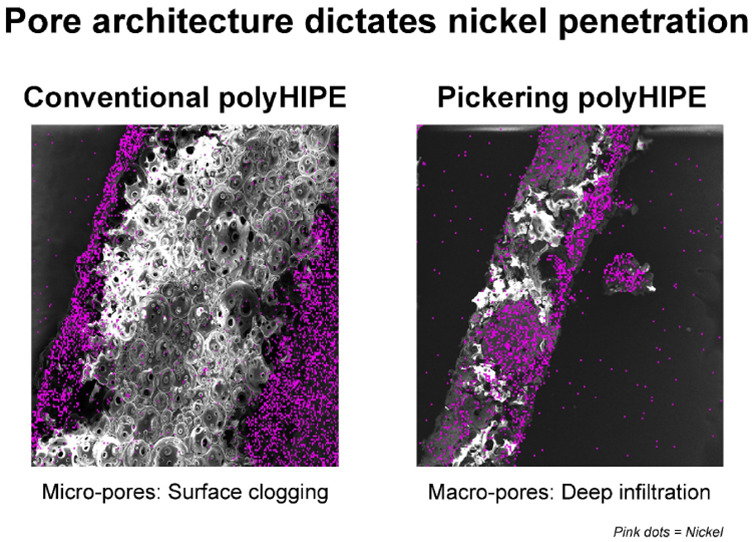

## 1 Introduction

Porous polymers have played a significant role in industrial and scientific applications for several decades. However, since 1990s, interest in these materials has grown significantly due to the development of techniques that enable control over both their pore architecture and chemical compositions.^1,2^ These materials have become important in a wide range of applications including adsorption, separation, tissue engineering, and energy storage.^3^

A critical design variable in porous polymers is the pore size and distribution. Tailoring the pore size enables optimization for different applications. For instance, a narrow, well-defined pore size distribution can improve selectivity and efficiency by offering precise control over parameters such as permeability, mechanical strength, and adsorption capacity.^4^ On the other hand, a wider range of pore sizes, encompassing both macro- and micropores, can enhance performance in filtration, where the ability to trap particles across a range of sizes is advantageous.^2^ Additionally, pores may be classified as open or closed: open pores form interconnected networks that facilitate fluid flow and diffusion, whereas closed pores are isolated and primarily contribute to insulation and enhanced mechanical strength.^5^

Several conventional methods are available for fabricating porous polymers, such as foaming, phase separation, electrospinning, freeze-drying, sacrificial templating, and replica templating.^5,6^ Despite their utility, many of these approaches face difficulties in providing consistent control over porosity, especially in generating well-organized, multiscale structures.^7^ In contrast, emulsion templating has emerged as a highly adaptable method capable of producing materials with open-cell structures, high porosity, and tunable pore sizes.^8^

PolyHIPEs are obtained by polymerizing high internal phase emulsions (HIPEs), which are typically defined as emulsions in which the internal phase constitutes more than 74% of the total volume. These biphasic systems consist of immiscible liquids, where the internal phase forms discrete droplets within a continuous phase.^9^ Upon curing, the continuous phase of HIPEs cross-links into a porous material with interconnected voids that replicate the aqueous droplet arrangement of the HIPE template.

A significant advantage of emulsion templating is the ability to manipulate pore morphology by altering the HIPE’s composition and the stabilization mechanism employed during HIPE preparation.^10–13^ Stabilization can be achieved using either traditional surfactants^14,15^ or colloidal particles.^16–18^ The choice between these two has a profound effect on the resulting porous structure: surfactants tend to produce finer, more uniform pores, while particle-stabilized (Pickering) HIPEs often generate more heterogeneous pore structures.^17,19^

Recently, polyHIPEs have gained attention as promising templates for metal deposition.^20–25^ When coated with metals, such as via electroless nickel plating, these porous frameworks can be transformed into lightweight, conductive materials with hierarchical structures. These materials combine the lightweight nature and porosity of polymer scaffolds with the functional advantages of metallic surfaces, making them ideal for applications in electrocatalysis,^26,27^ electromagnetic interference (EMI) shielding,^28,29^ and gas diffusion electrodes.^30–32^ Nevertheless, achieving uniform coating throughout the porous network remains technically challenging.^24^ Factors like poor wettability of the internal surfaces, limited penetration of plating solutions, and inconsistent nucleation can hinder coating uniformity and performance. Metallized porous polymers prepared via HIPE templating have shown promising performance in CO_2_ conversion, due to their hierarchical pore structure and accessible active sites.^22^ In addition to polyHIPEs, other porous polymer systems, synthesized via alternative phase separation routes, have also been metallized with nickel for functional applications such as catalysis.^23^ Nickel-coated porous polymer frameworks have also been explored as efficient, recyclable catalysts, where hierarchical porosity enhances active site accessibility and catalytic performance.^33^ These studies highlight the importance of pore structure design in developing functional metal–polymer hybrid systems. Yet, the effect of emulsion stabilization on pore morphology and its impact on plating behavior in polyHIPEs remains largely unexplored.

Among the many variables that influence metallization quality, the pore morphology of the polyHIPE template is particularly significant. Larger pores allow for better infiltration of metal ions during plating, thereby supporting deeper and more uniform coatings.^34,35^ Smaller pores, while offering increased surface area, may obstruct solution flow and reduce coating efficiency.^36,37^ Accordingly, understanding the relationship between pore structure and coating behavior is essential for optimizing the fabrication of advanced porous metal-based structures.

In this study, inherently porous polyHIPE thin films, where the HIPE was stabilized either by Hypermer B246 or by IBOA particles, were employed as templates for electroless nickel plating. It has been previously demonstrated that Hypermer B246 and IBOA microparticles can independently stabilize water-in-oil (W/O) emulsions in systems where the oil phase consists of EHA/IBOA/TMPTA.^17^ In this context, Hypermer B246 functions as a conventional molecular surfactant whereas IBOA microparticles act as Pickering stabilizers. Hypermer B246 was selected as a standard polymeric surfactant to generate a conventional, fine-pored network, whereas IBOA-based microparticles were utilized to produce a Pickering emulsion with a distinctly larger and more heterogeneous macropore architecture. By keeping the base monomer composition constant, the intended comparison between these two fundamentally different stabilization mechanisms aims to isolate the effect of pore morphology on mass transport and metal infiltration. The obtained polyHIPEs were sectioned into thin films and subsequently metallized to evaluate the influence of pore morphology on coating behavior. Particular attention was given to the effect of stabilizer type on the resulting pore architecture, and consequently, the uniformity, penetration depth, and overall efficiency of nickel deposition within the porous network.

## 2 Materials and Methods

### 2.1 Materials

2-Ethylhexyl acrylate (EHA), isobornyl acrylate (IBOA), trimethylolpropane triacrylate (TMPTA), potassium persulfate (KPS), benzoyl peroxide (BPO), 3-(trimethoxysilyl)­propyl methacrylate, tin­(II) chloride (SnCl_2_), palladium­(II) chloride (PdCl_2_), boric acid (H_3_BO_3_), and ∼37% hydrochloric acid (HCl) were all purchased from Sigma-Aldrich. The surfactant Hypermer B246-SO-M was donated by Croda. Electroless nickel plating solutions (Part A and Part B) were purchased from Caswell UK. All chemicals were used as received without further purification.

### 2.2 Methods

#### 2.2.1 IBOA-Based Microparticle Synthesis

IBOA-based microparticles were synthesized via ultrasound-assisted oil-in-water (o/w) precipitation polymerization. The organic internal phase was composed of 75 wt % IBOA and 25 wt % TMPTA. A total of 1 g of this monomer blend was added to 9 g of an aqueous solution containing 0.5 wt % Tween 20 and 2 wt % KPS as a thermal initiator (relative to organic phase). The mixture was emulsified using a probe sonicator (Hielscher UP100H) at 100 W, 30 kHz for 1 min. The resulting emulsion was transferred to a convection oven and polymerized at 65 °C for 18 h. Postpolymerization, particles were washed with methanol, centrifuged (14,000 rpm, 15 min), redispersed in deionized water via sonication, and dried overnight at 65 °C. The synthesis steps of IBOA-based microparticles are illustrated in Figure 1. The synthesized microparticles were previously characterized by our group.^38^ Briefly, these particles possess an average diameter of approximately 200 nm and a water contact angle of ∼82°.

**Figure 1:**
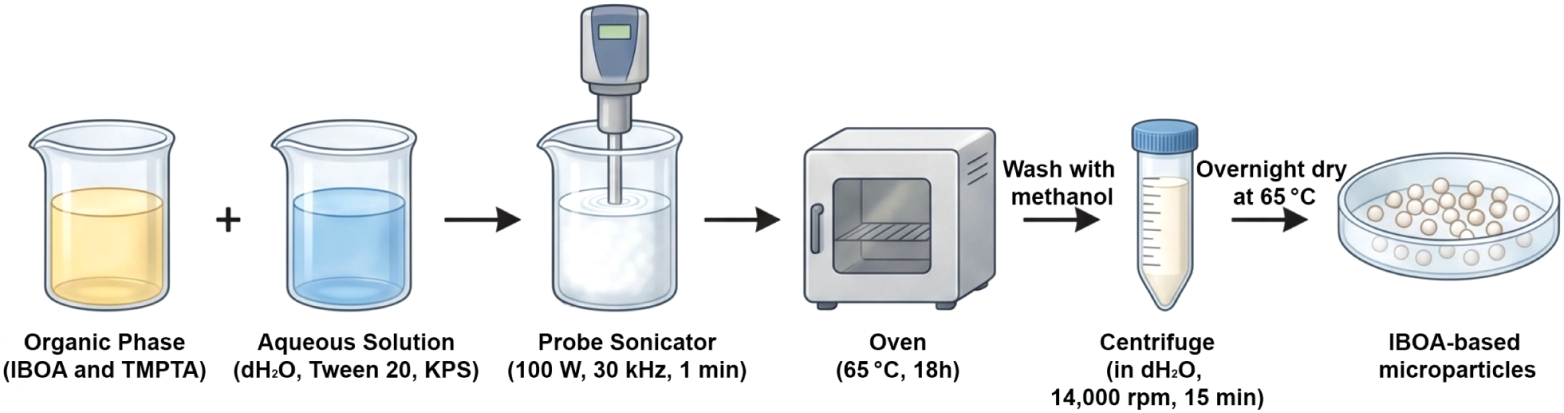
Synthesis steps of IBOA-based microparticles (created using illustrae.co and modified using Adobe Photoshop CC 2015).

#### 2.2.2 Preparation of polyHIPE Thin Films

PolyHIPEs were fabricated using a monomer mixture comprising 63 wt % EHA, 21 wt % IBOA, and 16 wt % TMPTA, with a total monomer content of 4 g. Stabilizers (3 wt %), either IBOA microparticles or Hypermer B246 surfactant, were incorporated into the monomer mixture. For particle-stabilized systems, the dispersion was facilitated by 1 min sonication, whereas Hypermer B246 was dissolved in the monomer mixture through mild heating and shaking. Subsequently, BPO (0.1 g per 4 g of monomer) was added to complete the continuous phase of the emulsion. The internal aqueous phase (dH_2_O, 26.6 mL) was added dropwise using a syringe pump (0.8 mL/min) while stirring at 500 rpm with an overhead stirrer to prepare a water-in-oil (W/O) HIPE with an internal phase volume fraction of 85 vol %. The resulting HIPEs were transferred into 10 mL syringes and placed in a convection oven at 65 °C for 18 h to induce polymerization to obtain cylindrical polyHIPEs. The cured polyHIPEs were then removed from the syringes and further dried at 60 °C overnight. Finally, 300 and 600 μm polyHIPE thin films were sectioned using a vibratome (5100 mz, Campden Instruments). Fabrication steps of Pickering and conventional polyHIPEs are presented in Figure 2.

**Figure 2:**
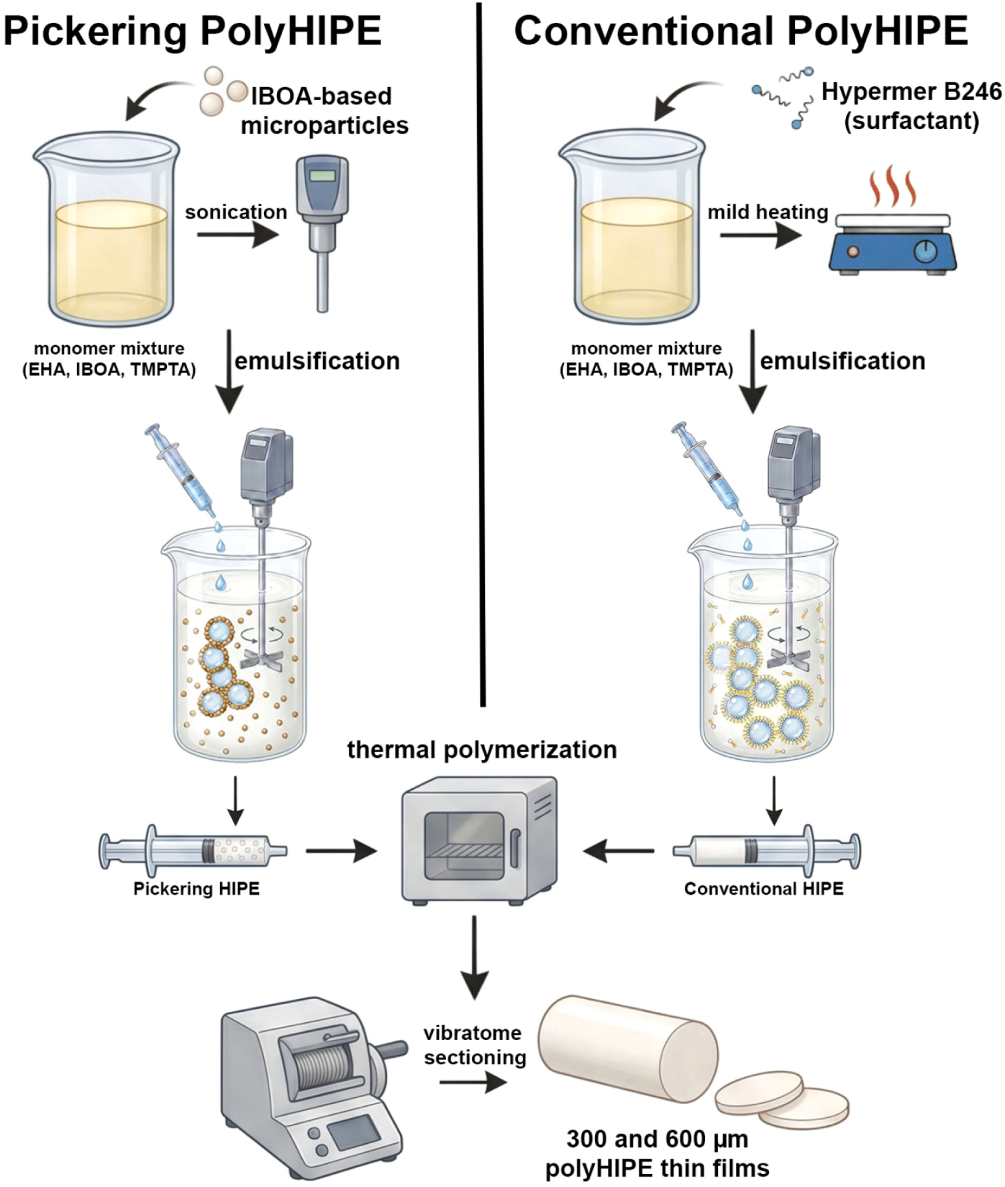
Fabrication steps of Pickering and conventional polyHIPEs (created using illustrae.co and modified using Adobe Photoshop CC 2015).

To improve clarity, polyHIPEs obtained from Hypermer B246-stabilized HIPEs are referred to as “conventional polyHIPEs”, whereas those derived from IBOA microparticle-stabilized HIPEs are referred to as “Pickering polyHIPEs” throughout this article.

#### 2.2.3 Metallization of polyHIPE Thin Films

The nickel plating procedure, along with the pretreatment steps established in our earlier study, was applied in this work.^24^ A schematic representation of the full process is provided in Figure 3. Prior to metallization, the polyHIPE surfaces were functionalized to enhance hydrophilicity, and activation treatments were performed to promote effective nickel deposition onto the polymer substrate.

**Figure 3:**
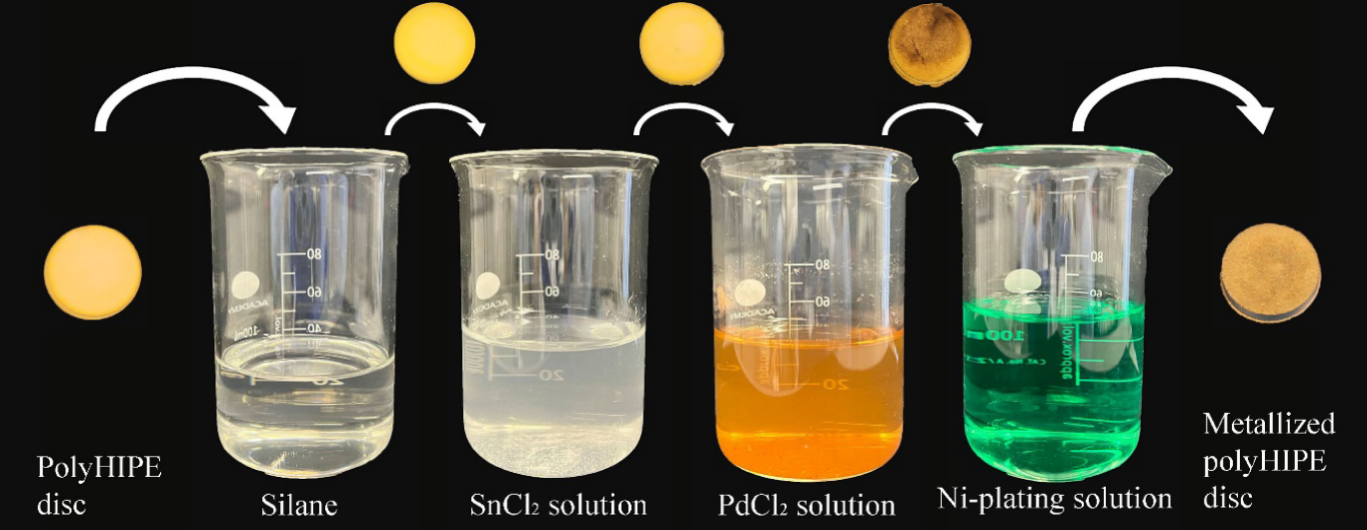
Schematic diagram of the electroless nickel plating process. Figure taken with permission from American Chemical Society, copyright 2023.^24^

Initially, polyHIPE thin films were degassed in a vacuum oven for 6 h to eliminate trapped gases within the porous network. Following this step, the samples were immersed in 3-(trimethoxysilyl)­propyl methacrylate (TMPSM) at 40 °C for 5 min to improve surface hydrophilicity. The silanized films were subsequently dried at 65 °C in a conventional oven for 4 days until complete dryness was achieved.

After drying, the silanized thin films were sequentially immersed in SnCl_2_ and PdCl_2_ activation baths at 40 °C for 20 and 10 min, respectively. Between the immersion steps, excess liquid was gently removed by blotting the films with absorbent tissue paper, specifically avoiding any squeezing to prevent mechanical damage to the porous network. The SnCl_2_ solution was prepared by dissolving 0.8 wt % SnCl_2_ in 100 mL of distilled water, the pH of which was adjusted to 1.2 by the dropwise addition of HCl. The PdCl_2_ solution was composed of 0.06 wt % PdCl_2_, 2 wt % H_3_BO_3_, and 100 mL of distilled water, with its pH similarly adjusted to 1.7 by adding HCl dropwise. A volume of 100 mL of each solution was used to treat a batch of 9 polyHIPE thin films to ensure clean and consistent surface activation. In this two-step activation process, Sn^2+^ ions are first adsorbed onto the polymer surface (sensitization) to act as reducing sites for Pd^2+^ ions. Subsequently, Pd^2+^ ions are reduced to metallic palladium nuclei (activation), which serve as catalytic seeds for the autocatalytic deposition of nickel.

Finally, the electroless nickel plating bath was prepared using Caswell’s commercial solutions, comprising 5 vol % Part A, 15 vol % Part B, and 80 vol % dH_2_O. The surface-activated polyHIPE thin films were immersed in the plating bath at 90 °C for 30 min. The concentrations of each component used in the metallization process are detailed in Table 1.

**Table 1:** Concentrations of Pretreatment and Electroless Nickel Plating Solutions

								Electroless nickel plating solution (Caswell)		
Silane	SnCl_2_ solution (pH: 1.2)			PdCl_2_ solution (pH: 1.7)				Part		
TMPSM	SnCl_2_	HCl	dH_2_O	PdCl_2_	H_3_BO_3_	HCl	dH_2_O	A	B	dH_2_O
100%	0.8 wt %	5 drops	100 mL	0.06 wt %	2 wt %	8 drops	100 mL	5 vol %	15 vol %	80 vol %

#### 2.2.4 Characterization

##### 2.2.4.1 Scanning Electron Microscopy/Energy Dispersive X-ray Analysis (SEM/EDX)

The internal morphology of both nonmetallized and metallized polyHIPE samples, as well as the surface features of the metallized specimens, was examined using a FEI Inspect F SEM. Before imaging, nonmetallized samples were gold coated to enhance conductivity. All SEM imaging was performed at an accelerating voltage of 5 kV. ImageJ software was used to quantify pore sizes and nickel layer thicknesses. The average pore size was calculated by measuring and averaging 150 pores. To account for dimensional underestimation due to nonideal sectioning angles, a correction factor (2/√3) was applied to the pore size measurements.^39^

Elemental composition of the metallized structures was assessed using SEM (Inspect F, FEI) with an energy dispersive spectrometer with 20 kV power.

##### 2.2.4.2 X-ray Diffraction (XRD)

X-ray diffraction was used to analyze the crystallographic and phase structure of the metallized polyHIPE thin films. Measurements were conducted using a PANalytical Aeris, operating at the reduced fluorescence measurement method (Cu tube 30 kV 40 mA, 1/4° divergence slit, a 0.15 mm Ni filter, 0.02 Rad soller slits). Diffraction data were recorded over a 2θ range of 0° to 100°, using a step size of 0.02°.

##### 2.2.4.3 Helium Pycnometry

The bulk density of cylindrically shaped nonmetallized and metallized polyHIPE samples was calculated by dividing the measured mass by the geometrically determined volume. Skeletal density measurements were performed using a gas pycnometer (AccuPyc 1340, Micromeritics). The porosity (*P*_Ø_) of the samples was then calculated using the following equation

1 $$P_{Ø}=\left(1-\frac{\rho_{\mathrm{sd}}}{\rho_{c}}\right)\times 100$$

where ρ_sd_ is the skeletal density, and ρ_c_ is the calculated density.

##### 2.2.4.4 Statistical Analysis

All statistical analyses were performed using OriginPro 2023. Mean values, standard deviations, and statistical significance were determined using one-way ANOVA followed by Tukey’s multiple comparison test. A *p*-value of 0.05 or lower was considered indicative of a statistically significant difference. Each analysis was performed with three replicates (*n* = 3), as noted in the respective figure and table captions.

## 3 Results and Discussion

### 3.1 Porous Structure of polyHIPEs

A comprehensive analysis of the pore microstructure and size distribution of conventional (H-stabilized) and Pickering (IBOA-stabilized) polyHIPEs are presented in Figure 4. This figure illustrates qualitative and quantitative evaluations using SEM images (Figure 4A and B) and statistical analysis (Figure 4C–E) to demonstrate the impact of stabilizer on pore morphology.

**Figure 4:**
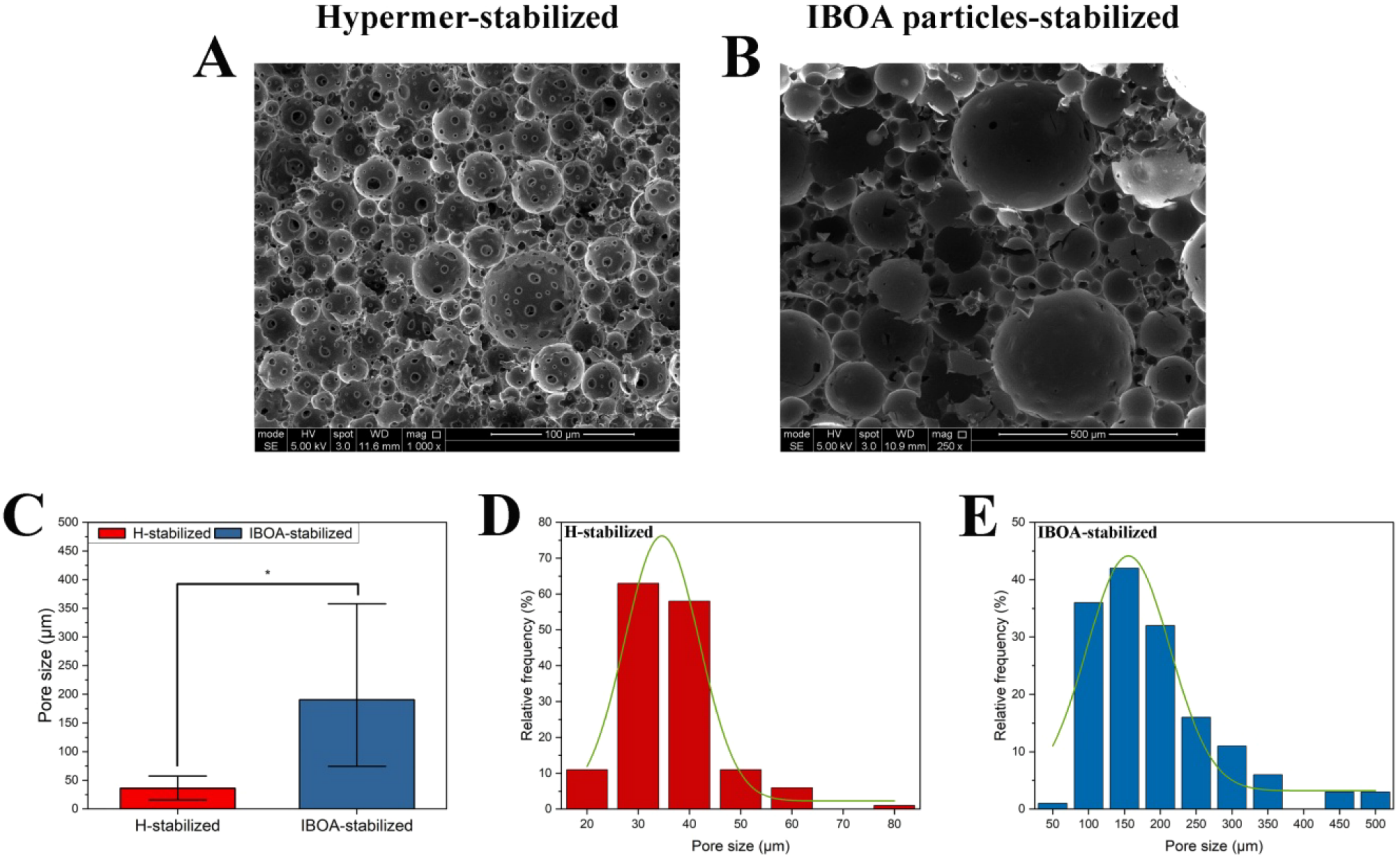
SEM images presenting porous microstructure of (A) conventional and (B) Pickering polyHIPEs. (C) Pore sizes showing statistical differences and pore size distributions of (D) conventional and (E) Pickering polyHIPEs. *: significant difference (*p* < 0.05) and *n* = 3. (Hypermer-stabilized HIPEs: conventional polyHIPEs, Hypermer-stabilized, H-stabilized and IBOA microparticle-stabilized HIPEs: Pickering polyHIPEs, IBOA particles-stabilized, IBOA-stabilized).

The average pore sizes of conventional and Pickering polyHIPEs are 36.24 ± 9.43 μm and 190.64 ± 87.77 μm, respectively (Table 2). As shown in Figure 4C, there is a statistically significant difference in pore sizes between the conventional and Pickering polyHIPEs, confirming the influence of the stabilizer type on pore morphology. This observation is consistent with previous findings, which indicate that HIPEs stabilized by surfactants at concentrations similar to those of particles result in smaller pore sizes.^17,19^ Furthermore, the histograms in Figure 4D and Figure 4E illustrate the distribution of pore sizes for conventional and Pickering polyHIPEs, respectively. The histogram for conventional polyHIPEs demonstrated a relatively narrow distribution with a peak between 20–80 μm, indicating the uniformity of the pores. Conversely, the histogram for Pickering polyHIPEs showed a broader and more skewed distribution, with a notable shift toward larger pore sizes, ranging from approximately 50 to over 500 μm. The pore distribution curve suggests a wider variance in pore size, which aligns with the SEM observations of increased coalescence and irregularity in the Pickering polyHIPE. The observed pore size distribution can be attributed to the stability of the emulsion during polymerization. The low dispersity observed in the conventional polyHIPE suggests that the emulsion remained stable throughout polymerization, whereas coalescence of emulsion droplets, likely due to elevated temperatures, was evident in Pickering polyHIPE.

**Table 2:** Porosity (%), Pore Size (μm), and Density (g/cm^3^) Values of the Nonmetallized and Metallized polyHIPE Thin Films (*n* = 3)^a^

Samples	Pore size (μm)	Porosity (%)	Density (g/cm^3^)
Conventional polyHIPE	36.24 ± 9.43	85.57 ± 0.34	0.20 ± 0.002
Ni_Conventional polyHIPE	n/a	80.58 ± 0.17	0.31 ± 0.014
Pickering polyHIPE	190.64 ± 87.78	85.33 ± 0.16	0.16 ± 0.006
Ni_Pickering polyHIPE	n/a	80.75 ± 0.34	0.35 ± 0.019

a Hypermer-stabilized HIPE: conventional polyHIPE, nickel coated polyHIPE derived from hypermer-stabilized HIPE: Ni_Conventional polyHIPE, IBOA microparticle-stabilized HIPE: Pickering polyHIPE, nickel coated polyHIPE derived from IBOA microparticle-stabilized HIPE: Ni_Pickering polyHIPE.

### 3.2 Metallization of polyHIPE Thin Films

Figure 5 presents a comprehensive analysis of the metallization process applied to conventional and Pickering polyHIPE thin films. The figure combines macroscopic and microscopic observations (Figure 5A–D) with compositional (Figure 5E) and structural (Figure 5F) analyses, illustrating the effect of pore morphology on the deposition and crystallinity of nickel coatings.

**Figure 5:**
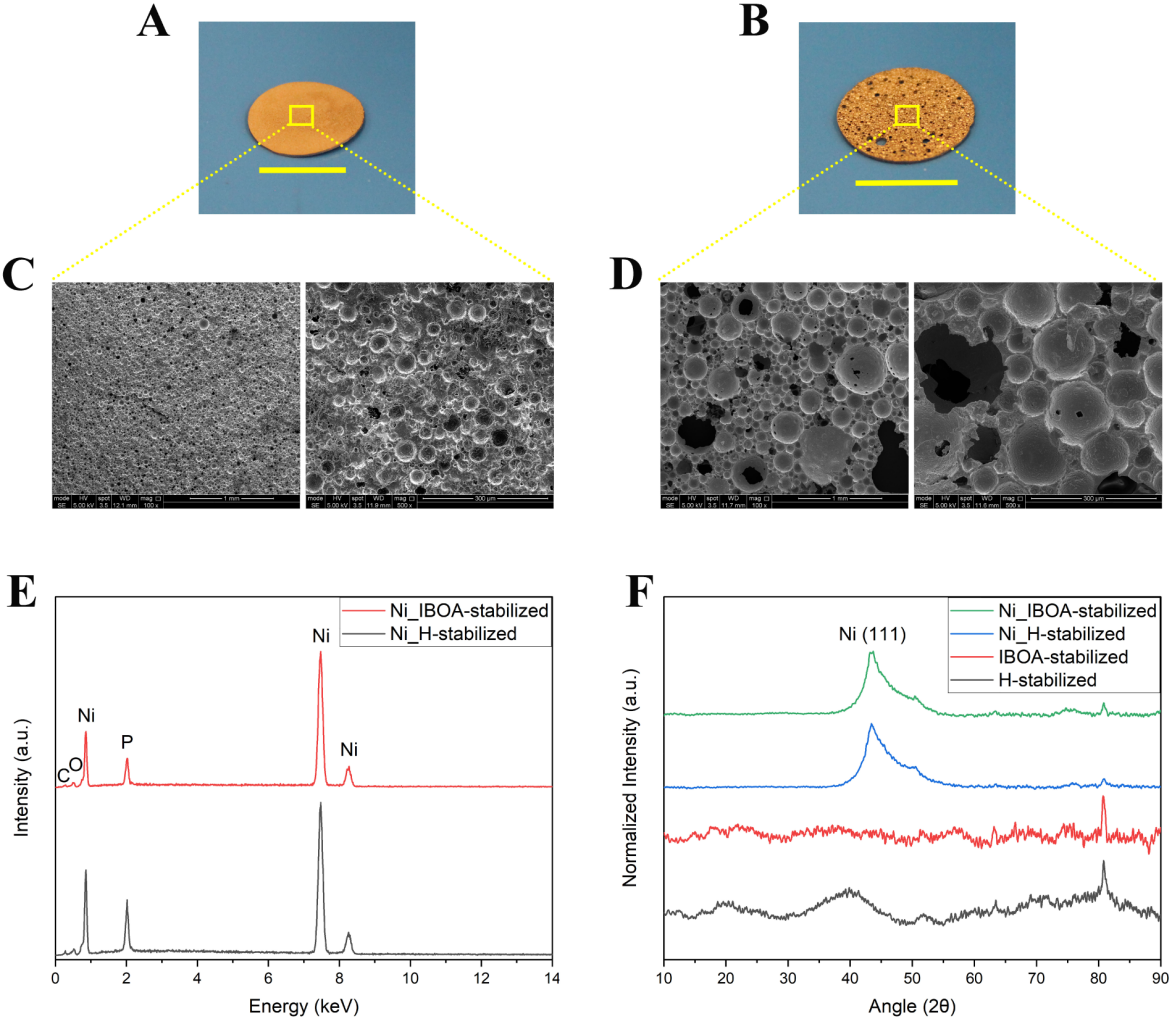
Metallized (A) conventional and (B) Pickering polyHIPE thin films (scale bars: 10 mm) and (C, D) their SEM images, respectively. (E) EDX and (F) XRD analyses of the metallized polyHIPE thin films. (Nickel coated polyHIPEs derived from Hypermer-stabilized HIPEs: metallized conventional polyHIPEs, Ni_H-stabilized and IBOA microparticle-stabilized HIPEs: metallized Pickering polyHIPEs, Ni_IBOA-stabilized).

Figure 5A and B display the physical appearance of metallized conventional and Pickering polyHIPE thin films, respectively. As expected, both exhibited a metallic appearance. The images demonstrated a clear contrast in surface morphology, with the conventional polyHIPEs appearing smoother, whereas the Pickering polyHIPEs exhibited a rougher and more porous texture. The difference in macroscopic appearance correlates with the inherent pore size and distribution, emphasizing the role of stabilizer choice in determining the final surface characteristics of polyHIPEs. The visible porosity in metallized Pickering polyHIPE thin films stems from the larger pores.

Figure 5C and D provide SEM micrographs of the metallized thin films, offering insight into the structural details at the microscopic level. The SEM images of conventional polyHIPEs (Figure 5C) reveal a fine porous network with relatively smaller and more homogeneously distributed pores. While absolute macroscale continuity cannot be solely verified via surface SEM, the presence of a conformal and widespread metallic morphology, supported by the strong crystalline Ni signals in the XRD and EDX spectra (Figure 5E and F), indicates effective metallization with substantial nickel coverage. This controlled pore structure likely facilitates uniform metal deposition by providing a consistent surface area for electroless plating. In contrast, the SEM images of Pickering polyHIPEs (Figure 5D) illustrate a highly porous structure with significantly larger and more irregularly distributed pores. The metallization process appears less uniform. This variability may be attributed to the larger pore size, which can lead to nonuniform deposition and reduced connectivity between metal-coated regions. The increased pore size, however, may enhance the potential for metal infiltration, a characteristic advantageous for applications requiring high permeability, such as catalysis.^40^

EDX analysis presented in Figure 5E, along with the XRD analysis shown in Figure 5F, provided further evidence of successful nickel plating. The characteristic Ni peaks observed prominently in both spectra, indicating successful metallization of both conventional and Pickering structures. Additional peaks corresponding to phosphorus (P) are visible, which is a common feature in electroless nickel plating, where phosphorus acts as a codeposited element that influences the mechanical and electrical properties of the metal layer.^41^ The presence of oxygen (O) in the spectra suggests partial oxidation of the metal layer.^41^ It should be noted that traces of the activation metals (Sn and Pd) were not detected in the EDX spectra. This is attributed to the relatively thick nickel overlayer, which masks the underlying monolayer-scale seed particles, preventing their detection by surface-sensitive EDX mapping.

X-ray diffraction (XRD) patterns of both metallized and nonmetallized polyHIPEs highlight the crystalline characteristics of the deposited nickel. The diffraction peaks corresponding to the face-centered cubic (FCC) Ni (111) plane are distinctly observed in the metallized samples at 2θ = 44.5°, which is in good agreement with the standard reference database (JCPDS card No. 04-0850), confirming the formation of a metallic nickel phase.^24,42^ The XRD patterns of the nonmetallized samples exhibited broad features characteristic of amorphous polymeric materials, consistent with the expected structural properties of polyHIPEs. The emergence of crystalline peaks upon metallization confirms the successful deposition of nickel to the polyHIPE templates.

Furthermore, cross-section SEM images of metallized polyHIPE thin films having various thicknesses (300 μm conventional polyHIPE, 300 μm Pickering polyHIPE, and 600 μm Pickering polyHIPE) along with corresponding EDX Ni-mapping are presented in Figure 6A–C. This figure illustrates the internal structure and elemental distribution, clearly demonstrating how deep nickel penetrates into polyHIPE thin films of varying thicknesses.

**Figure 6:**
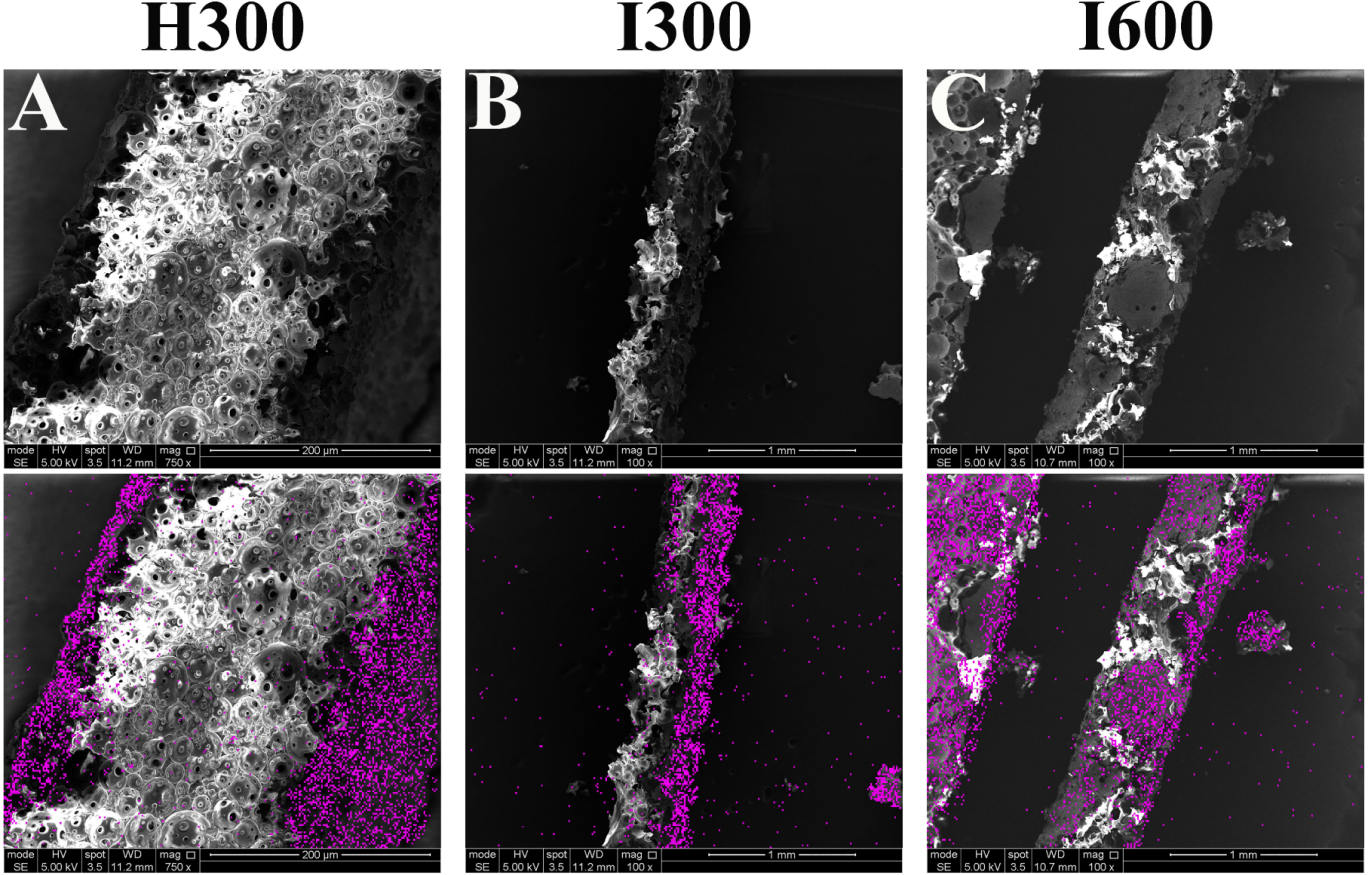
Cross-section SEM images of the metallized polyHIPE thin films and their corresponding EDX analysis (purple: nickel mapping). (A) 300 μm conventional (H300), (B) 300 μm Pickering (I300), and (C) 600 μm Pickering (I600) polyHIPE thin films. (H300:300 μm polyHIPE thin films derived from Hypermer-stabilized HIPEs, I300:300 μm polyHIPE thin films derived from IBOA microparticle-stabilized HIPEs, and I600:600 μm polyHIPE thin films derived from IBOA microparticle-stabilized HIPEs).

The cross-sectional SEM image and corresponding EDX mapping of the 300 μm conventional polyHIPE (H300) (Figure 6A) reveal a highly porous and interconnected architecture with small, uniformly distributed pores. Nickel deposition was observed along the pore walls, indicating effective surface-level infiltration. However, the limited pore dimensions restricted metal diffusion, resulting in surface-dominated metallization. In contrast, the 300 μm Pickering polyHIPE (I300) (Figure 6B) exhibits substantially larger and more irregular pore structures. This morphology facilitated deeper nickel penetration, as confirmed by EDX mapping, which indicates near-complete metallization throughout the cross-section. To further evaluate the extent of metal infiltration in thicker architectures, a 600 μm Pickering polyHIPE (I600) was analyzed. The 600 μm sample (Figure 6C) also demonstrates enhanced nickel penetration, approaching near-complete coverage. These results reinforce the concept that sufficiently large pores facilitate effective metal diffusion, enabling complete metallization within the porous network. While earlier studies have suggested the potential for internal metallization of polyHIPE structures, the EDX mapping presented here offers additional experimental confirmation of this capability.^20,21,24^

The overall porosity of the nickel-coated samples was approximately 80%, while the nonmetallized polyHIPEs exhibited a higher porosity of around 85%, consistent with the internal phase ratio used (Table 2). This decrease in porosity following metallization is expected and indicates successful nickel deposition within the pore network. Supporting this, the skeletal density increased from 0.20 g/cm^3^ to 0.31 g/cm^3^ for conventional polyHIPE samples and from 0.16 g/cm^3^ to 0.35 g/cm^3^ for Pickering polyHIPE samples, reflecting the incorporation of nickel into the polymer framework.

Although Pickering polyHIPEs exhibited restricted interconnectivity (qualitatively defined by the noticeable scarcity of visible pore throats between adjacent macroscopic voids in the SEM micrographs) compared to the highly perforated conventional polyHIPEs, they exhibited greater nickel penetration. Notably, the noninterconnected morphology observed in the Pickering HIPE templates contrasts with our previous findings, in which Pickering polyHIPEs exhibited an interconnected porous structure.^9,17,38^ In those earlier studies, this interconnectivity was attributed to the arrested coalescence of emulsion droplets, where pore throats formed at the necking regions between adjacent droplets. In the present study, however, polymerization was conducted using a thermal initiator at elevated temperatures rather than a photoinitiator at room temperature. This observation offers new insight into the potential influence of polymerization temperature and/or the type of initiator on polyHIPE morphology. Despite this restricted interconnectivity, the Pickering polyHIPEs achieved superior internal metallization compared to the highly interconnected conventional samples. This phenomenon highlights the dominance of macropore diffusion over interconnectivity in this specific system. In conventional polyHIPEs, although highly interconnected, the small pore sizes (∼36 μm) likely led to rapid surface sealing; as nickel deposited on the outer pore walls, the entry pathways clogged before the solution could diffuse inward. In contrast, the massive pores in Pickering samples (∼190 μm) remained open long enough to sustain the mass transport of the plating solution into the deep matrix, despite the tortuous pathways. Additionally, it is hypothesized that the capillary pressure against fluid entry is significantly higher in the small pores of conventional polyHIPEs, which, combined with the hydrophobic nature of the polymer, may have further restricted the penetration of the aqueous plating solution. Future studies could experimentally verify this effect. Overall, this finding represents a promising step toward the fabrication of fully metal-coated, ultraporous polymeric structures.

Collectively, pore size plays a critical role in the metallization process of polyHIPE thin films, directly influencing metal infiltration, coating uniformity, and overall material performance. Larger pores, such as those observed in Pickering polyHIPEs, offer distinct advantages in applications requiring deep metal penetration and enhanced mass transport. The increased pore dimensions facilitate the diffusion of metal ions into the internal structure during electroless plating, potentially reducing the risk of surface-heavy deposition that often limits conductivity in thicker films. Additionally, larger pores can accommodate higher metal loading without excessively blocking interconnectivity, making them particularly suitable for catalytic applications where active metal sites need to be accessible for reactants. This advantage positions Pickering polyHIPEs as promising candidates for functional materials requiring controlled permeability and efficient metal integration, such as gas diffusion electrodes or structured catalytic supports.

## 4 Conclusion

This study demonstrates the influence of emulsion stabilization strategy on pore morphology and its impact on the electroless nickel plating behavior of polyHIPE thin films. Conventional polyHIPEs, with smaller and more uniform pores, supported surface-level metallization, whereas Pickering structures enabled deeper nickel infiltration due to their larger pore sizes. Although Pickering structures exhibited restricted interconnectivity compared to conventional polyHIPEs, their macroscopic pore dimensions facilitated deep mass transport, enabling uniform internal metallization. These findings demonstrate that pore size magnitude can override interconnectivity limitations in diffusion-controlled plating processes, preventing the surface sealing effect observed in smaller-pore systems. Cross-sectional SEM and EDX analyses confirmed that thicker Pickering films exhibited near-complete metallization in accessible regions, with some diffusion-limited areas. These results offer new insight into the structure–function relationship in metallized polyHIPEs and suggest that tuning pore morphology via stabilizer selection is a viable strategy for optimizing metal–polymer hybrid materials, particularly for applications requiring high metal accessibility and controlled porosity. Additionally, the current study raises new questions for future research, including the evaluation of the effects of temperature and/or the type of polymerization on pore throat formation in Pickering polyHIPEs via arrested coalescence, as well as the role of surface wettability in determining the efficacy of surface coatings.
